# SOCIAL CHARACTERISTICS OF RACIAL MINORITY STROKE SURVIVORS AND PERCEIVED DISCRIMINATION: A SECONDARY ANALYSIS

**DOI:** 10.1093/geroni/igad104.2473

**Published:** 2023-12-21

**Authors:** Lilcelia Williams, Jordann Antoan, Tina Harris, Joy Hammel, Natalie Leland, Elizabeth Skidmore

**Affiliations:** The University of Pittsburgh, Pittsburgh, Pennsylvania, United States; The University of Pittsburgh, Pittsburgh, Pennsylvania, United States; The University of Pittsburgh, Pittsburgh, Pennsylvania, United States; University of Illinois at Chicago, Chicago, Illinois, United States; The University of Pittsburgh, Pittsburgh, Pennsylvania, United States; University of Pittsburgh, Pittsburgh, Pennsylvania, United States

## Abstract

African American and Black persons experience significant disability after stroke. They also experience high external stressors, including discrimination, which may attenuate stroke recovery. Further understanding of the associations among external stressors, disability severity, and attenuated recovery are important to reduce healthcare disparities and improve patient outcomes. However, persons of the same race may not have the same lived experiences due to variations in social characteristics such as age or education. Associations between these social characteristics and perceived discrimination may offer insights into the recovery of older adult African American and Black stroke survivors. We conducted a secondary analysis of 11 poststroke patients who were recruited to participate in the Black Lived Experiences of Stroke Study (female=10, age=67±11, ≥2 years post-secondary education=63%) to assess the frequency of perceived discrimination. Perceived discrimination was measured with the Everyday Discrimination Scale survey. We examined correlations between age and education and perceived discrimination using Cramer’s V tests. Semi-structured participant interviews were also examined for examples of perceived discrimination during poststroke healthcare. Nine participants reported perceived discrimination post-stroke. Analyses revealed a clinically meaningful but not statistically significant relationship between chronological age and perceived discrimination (Cramer’s V=0.62, p=0.12). There was no meaningful relationship between educational level and perceived discrimination. All participants reported challenges with communication, missed diagnoses and poor delivery of care poststroke. Findings support the need for additional research to examine the relationships between participants’ social characteristics and perceived discrimination among Black and African American stroke survivors, and the impact on stroke recovery.

